# From Sea to Lab: Angiotensin I-Converting Enzyme Inhibition by Marine Peptides—Mechanisms and Applications

**DOI:** 10.3390/md22100449

**Published:** 2024-09-30

**Authors:** Du-Min Jo, Fazlurrahman Khan, Seul-Ki Park, Seok-Chun Ko, Kyung Woo Kim, Dongwoo Yang, Ji-Yul Kim, Gun-Woo Oh, Grace Choi, Dae-Sung Lee, Young-Mog Kim

**Affiliations:** 1 National Marine Biodiversity of Korea (MABIK), Seochun 33662, Republic of Korea; dmjo@mabik.re.kr (D.-M.J.); seokchunk@mabik.re.kr (S.-C.K.); kimkw79@mabik.re.kr (K.W.K.); dwyang@mabik.re.kr (D.Y.); jiyul2224@mabik.re.kr (J.-Y.K.); ogwchobo@mabik.re.kr (G.-W.O.); gchoi@mabik.re.kr (G.C.); daesung@mabik.re.kr (D.-S.L.); 2 Ocean and Fisheries Development International Cooperation Institute, Pukyong National University, Busan 48513, Republic of Korea; 3 International Graduate Program of Fisheries Science, Pukyong National University, Busan 48513, Republic of Korea; 4 Smart Food Manufacturing Project Group, Korea Food Research Institute, Wanju 55365, Republic of Korea; skpark@kfri.re.kr; 5 Department of Food Science and Technology, Pukyong National University, Busan 48513, Republic of Korea

**Keywords:** ACE inhibitors, marine peptides, cardiovascular diseases, mechanism of action, structural diversity, antihypertensive activity, molecular interactions, therapeutic potential

## Abstract

To reveal potent ACE inhibitors, researchers screen various bioactive peptides from several sources, and more attention has been given to aquatic sources. This review summarizes the recent research achievements on marine peptides with ACE-inhibitory action and application. Marine peptides are considered excellent bioactives due to their large structural diversity and unusual bioactivities. The mechanisms by which these marine peptides inhibit ACE include competitive binding to ACEs’ active site, interfering with ACE conformational changes, and avoiding the identification of substrates. The unique 3D attributes of marine peptides confer inhibition advantages toward ACE activity. Because IC_50_ values of marine peptides’ interaction with ACE are low, structure-based research assumes that the interaction between ACE and peptides increased the therapeutic application. Numerous studies on marine peptides focused on the sustainable extraction of ACE-inhibitory peptides produced from several fish, mollusks, algae, and sponges. Meanwhile, their potential applications and medical benefits are worth investigating and considering. Due to these peptides exhibiting antioxidant, antihypertensive, and even antimicrobial properties simultaneously, their therapeutic potential for cardiovascular disease and other illnesses only increases. In addition, as marine peptides show better pharmacological benefits, they have increased absorption rates and low toxicity and could perhaps be modified for better stability and bioefficacy. Biotechnological advances in peptide synthesis and formulation have greatly facilitated the generation of peptide-based ACE inhibitors from marine sources, which subsequently offer new treatment models. This article gives a complete assessment of the present state of knowledge about marine organism peptides as ACE inhibitors. In addition, it emphasizes the relevance of additional investigation into their mechanisms of action, the optimization of manufacturing processes, and assessment in in vivo, preclinical, and clinical settings, underlining the urgency and value of this study. Using marine peptides for ACE inhibition not only broadens the repertory of bioactive compounds but also shows promise for tackling the global health burden caused by cardiovascular diseases.

## 1. Introduction

Cardiovascular diseases (CVDs) continue to be a major issue for the health of people all over the globe since they are responsible for a considerable amount of morbidity and death. The presence of hypertension, which affects roughly one-third of individuals throughout the world, stands out as a significant risk factor among the many variables that are involved in the pathogenesis of CVDs [1,2]. Through its involvement in the renin-angiotensin–aldosterone system (RAAS), the angiotensin I-converting enzyme (ACE) is an essential component in the process of maintaining cardiovascular homeostasis and controlling blood pressure [3]. Angiotensin I is converted into angiotensin II, which is a powerful vasoconstrictor after being facilitated by ACE. There is a correlation between this process and the development of hypertension since it leads to an increase in peripheral resistance. The therapeutic suppression of ACE has been an essential component in the management of hypertension and other cardiovascular disorders and conditions connected to it [4]. ACE inhibitors, which include captopril and lisinopril, have significantly lowered blood pressure, improved cardiovascular outcomes, and reduced death rates. This has resulted in a revolution in the therapeutic landscape. The fact that traditional ACE inhibitors are linked with side effects such as dry cough and angioedema, despite the fact that they are clinically successful, highlights the continuous need for alternatives that are both safer and more effective [5]. Most recently, natural bioactive substances have been receiving a lot of interest as possible ACE inhibitors. Peptides produced from marine organisms have especially been the focus of this study. There is a great reservoir of biodiversity that may be found in marine environments, which includes a wide variety of peptides that each have their own distinctive structural characteristics and biological activity [6]. Marine peptides have gained a lot of interest because of their ability to block ACEs and their wider pharmacological potential, which includes antioxidant, antibacterial, and antihypertensive effects. The structural variety of marine peptides and the uniqueness of their interactions with biological targets are two reasons why they are so appealing.

Unlike synthetic drugs, marine peptides often have better bioavailability, reduced toxicity, and greater compatibility with biological systems [7,8]. Furthermore, improvements in biotechnological methods have made it possible to extract, purify, and modify these peptides in a sustainable manner, increasing their therapeutic potential [9]. The search for ACE inhibitors from marine species has turned up peptides from a variety of sources, including fish, mollusks, algae, and sponges [10]. These peptides differ in size, amino acid makeup, and structural features, influencing their bioactivity and mode of action [11]. ACE-inhibitory peptides may function by competitively inhibiting the active site of ACE, blocking the conversion of angiotensin I to angiotensin II [12].

Additionally, peptides may affect ACE activity by modifying enzyme structure or interfering with substrate recognition, opening new pathways for therapeutic intervention [13]. Structural investigations utilizing nuclear magnetic resonance (NMR) spectroscopy, X-ray crystallography, and molecular docking have shown the molecular connections between marine peptides and ACE. These investigations have shed light on the binding modalities, critical amino acid residues implicated in binding, and structural requirements for effective ACE inhibition [14]. Such mechanistic insights are critical for the rational design and development of peptide-based ACE inhibitors that have higher potency and selectivity. Aside from their function as ACE inhibitors, marine peptides have a wide range of pharmacological properties that might alleviate different CVD pathogenesis elements. For example, certain peptides have antioxidant capabilities that protect cardiovascular tissues from oxidative stress-induced damage [15,16]. Others have antihypertensive effects via regulating vascular tone or reducing endothelial dysfunction, which complements their ACE-inhibitory activities [17,18]. The translation of marine peptides from laboratory research to clinical applications has the potential to provide safer and more effective alternatives to traditional ACE inhibitors in CVD management. Clinical trials and preclinical research have shown that some marine-derived peptides may decrease blood pressure and improve cardiovascular function in animal models and humans. These results highlight the therapeutic potential of marine peptides as an adjunct therapy or alternative to traditional ACE inhibitors, especially in those who suffer extreme side effects or inadequate responses to current treatments [19,20].

**Figure 1:**
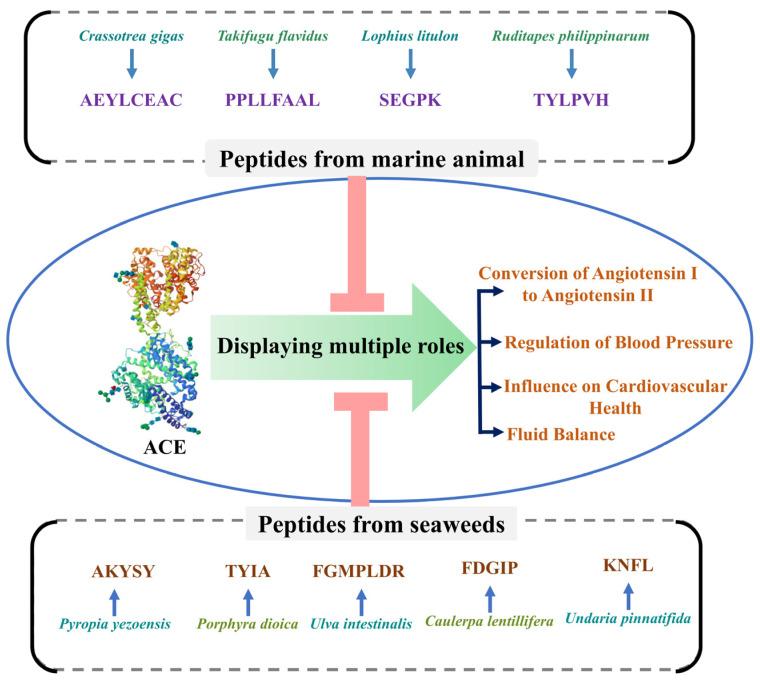
Bioactive peptides from marine organisms and their multifunctional roles in ACE inhibition and cardiovascular health.

This review provides a more focused and up-to-date examination of marine-derived ACE-inhibitory peptides, particularly highlighting recent advancements in extraction methods, structural analyses, and therapeutic applications. Although some of the previous review papers have reported on ACE inhibition by marine peptides [21,22], they have focused on land-based or synthetic ACE inhibitors. On the other hand, this review provides a more comprehensive and accurate examination of marine-derived ACE-inhibitory peptides. One of the most distinguishing features of this review is its emphasis on marine species, namely, mammals and seaweeds, as the sources of bioactive peptides. This article investigates the processes by which marine-derived peptides interact with the ACE enzyme using molecular docking experiments, demonstrating how these peptides bind to the ACE active sites and how their structural characteristics impact their bioactivity. This review seeks to collect existing information on marine organism peptides as ACE inhibitors, presenting molecular insights, highlighting recent advances, and addressing possible uses in cardiovascular medicine (Figure 1). Furthermore, the study highlights biotechnological breakthroughs that have improved the sustainable extraction, purification, and use of marine peptides, emphasizing their therapeutic potential not just as antihypertensive agents but also for wider cardiovascular health benefits. Marine peptides have the potential to significantly contribute to the therapy of hypertension and other cardiovascular disorders by bridging fundamental research and clinical practice, thereby enhancing patient outcomes and their quality of life. This review is separated into numerous parts: (1) a discussion of ACE structure and function, (2) a functional classification of peptides from various marine organisms as ACE inhibitors, (3) a mechanism of action supported by molecular, biochemistry, and molecular docking studies, (4) multiple potential biological roles, (5) a discussion of progress in in vivo and clinical studies, and (6) proposed future directions.

## 2. Angiotensin I-Converting Enzyme (ACE)

**Figure 2:**
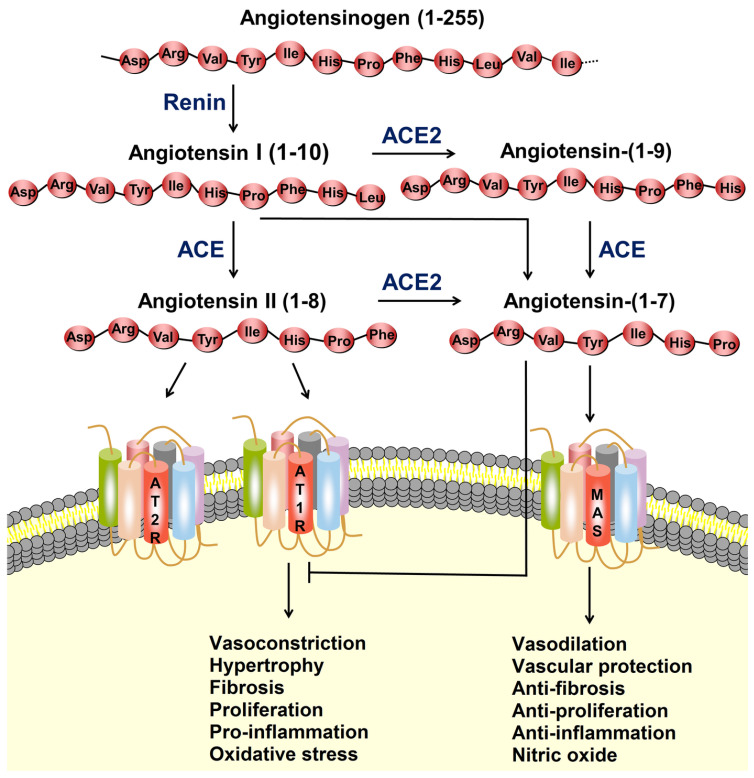
The renin–angiotensin system (RAS) and the ACE2/angiotensin-(1–7)/MAS axis. Renin converts angiotensinogen to angiotensin I (Ang I), which is then transformed into angiotensin II (Ang II) by angiotensin-converting enzyme (ACE). Ang II binds to the angiotensin type 1 receptor (AT1R), leading to fibrosis, vasoconstriction, proliferation, hypertrophy, oxidative stress, and inflammation. ACE2 converts Ang I and Ang II into angiotensin-(1–7), which then binds to the MAS receptor. This interaction promotes vasodilation and offers vascular protection, while also exhibiting anti-fibrotic, anti-proliferative, and anti-inflammatory effects. Furthermore, Ang II can bind to the angiotensin type 2 receptor (AT2R), which helps to counterbalance the effects mediated by the AT1R. Reprinted from [24], Copyright @ 2020, Springer Nature.

Angiotensin-I-converting enzyme (ACE; EC 3.4.15.1) is a zinc-dependent metallopeptidase belonging to the M2 family of dipeptidyl carboxypeptidases [23]. It plays a critical role in regulating blood pressure and electrolyte balance through its involvement in the renin–angiotensin system (RAS) and the kallikrein–kinin system (KKS) (Figure 2) [24]. Structurally, ACE is a single-chain glycoprotein with two functional domains: the N-domain and C-domain. Each domain contains a zinc-binding motif essential for its catalytic activity [25]. The active sites of ACE are responsible for cleaving two amino acids from the carboxyl terminus of specific peptides [26]. Functionally, ACE performs two main actions: it converts angiotensin I, an inactive decapeptide, into angiotensin II, a powerful vasoconstrictor, and it breaks down bradykinin, a peptide that acts as a vasodilator (Figure 2). These dual actions of ACE promote vasoconstriction and sodium retention, ultimately raising blood pressure. This makes ACE a critical enzyme in blood pressure regulation, as it directly influences vascular tone and fluid homeostasis [24].

ACE is a crucial enzyme within the RAS, a hormone network responsible for regulating blood pressure and fluid balance [27]. The RAS is activated when renin, an enzyme produced by the kidneys, transforms angiotensinogen, which is generated by the liver, into angiotensin I [28]. Subsequently, ACE converts angiotensin I into angiotensin II. This peptide is a powerful vasoconstrictor that raises blood pressure by narrowing blood vessels (Figure 2) [24]. Additionally, angiotensin II triggers aldosterone release from the adrenal glands, leading to increased sodium and water retention, which further raises blood volume and blood pressure [29]. Beyond blood pressure regulation, ACE also impacts the KKS by breaking down bradykinin, a peptide that promotes blood vessel dilation and lowers blood pressure. By inactivating bradykinin, ACE enhances its pressor effects, contributing to the complex control of vascular tone [30]. The renin–angiotensin–aldosterone system (RAAS) plays a vital role not only in blood pressure regulation but also in maintaining electrolyte balance and fluid homeostasis, making ACE inhibition a significant therapeutic strategy for cardiovascular and renal conditions [31].

In addition to ACE, a related enzyme called ACE2 plays a significant role in this system. While ACE raises blood pressure by forming angiotensin II, ACE2 helps counteract these effects by converting angiotensin II into angiotensin-(1–7), a peptide with vasodilatory and cardioprotective properties. Maintaining a proper balance between ACE and ACE2 is essential for homeostasis, and imbalances have been linked to various cardiovascular disorders [32,33].

Due to ACE’s pivotal role in hypertension, ACE inhibitors have become a mainstay in managing high blood pressure. These medications work by blocking ACE, thereby reducing the formation of angiotensin II, leading to lower blood pressure through decreased vasoconstriction and enhanced vasodilation [34]. Common ACE inhibitors such as captopril, lisinopril, enalapril, and ramipril are highly effective in treating hypertension, heart failure, and chronic kidney disease in at-risk patients [35].

The mechanism of ACE inhibitors involves competitive inhibition at the active site of the enzyme, which prevents angiotensin I from being converted into angiotensin II. Additionally, because ACE inhibitors block the degradation of bradykinin, they enhance the vasodilatory effects of this peptide, contributing to their antihypertensive action. However, the increased levels of bradykinin are also associated with side effects, such as dry cough and angioedema, which are common in patients using ACE inhibitors [5,36].

Despite their widespread use, synthetic ACE inhibitors have certain drawbacks. Some patients experience adverse reactions, including skin rashes, the loss of taste, and allergic responses. The most well-known side effect is a persistent dry cough, which can significantly affect patient compliance. More severe, though rare, adverse effects include angioedema, a potentially life-threatening condition [37]. To mitigate these issues, recent research has focused on developing new ACE inhibitors with fewer side effects or combining ACE inhibitors with other antihypertensive agents, such as angiotensin II receptor blockers (ARBs), which block the action of angiotensin II at its receptor without affecting bradykinin metabolism [38,39]. In addition to synthetic inhibitors, there is growing interest in natural ACE inhibitors derived from food sources, particularly from proteins found in marine materials [40,41]. For example, bioactive peptides from fish, such as skipjack roe and salmon by-products, have shown promising ACE-inhibitory activity without the adverse effects associated with synthetic drugs [42,43]. These natural peptides are being explored as alternatives to synthetic drugs due to their lower risk of side effects and their potential to provide additional health benefits, such as immune modulation and diabetic control [44,45].

Recent advances in peptide-based drug discovery have highlighted the potential of these natural inhibitors to provide a safer, more sustainable approach to managing hypertension. Furthermore, the ongoing exploration of marine resources as a source of bioactive peptides opens new avenues for the development of ACE inhibitors with novel mechanisms of action [46]. In light of the increasing demand for natural and sustainable therapies, these peptides represent a promising future direction in the treatment of hypertension and cardiovascular diseases [7,47].

## 3. Therapeutic Application of Inhibitory Peptides from the Different Marine Organisms and Their Action Mechanisms

**Figure 3:**
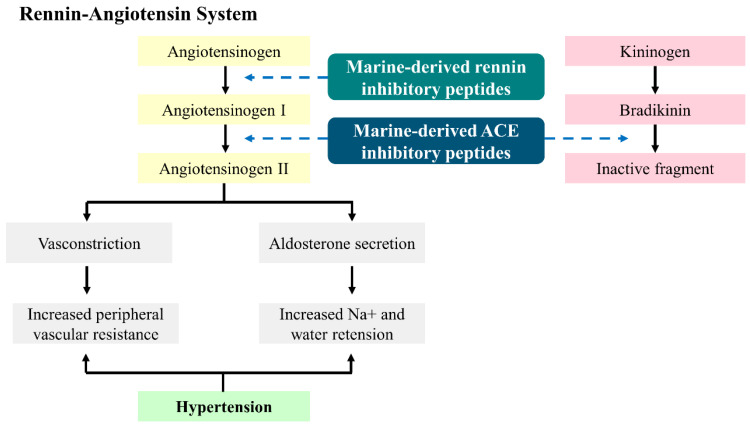
Inhibition of renin and angiotensin I-converting enzyme by marine-derived peptides. The figure has been redesigned based on the previous literature [51].

Marine organisms represent a vast and diverse source of bioactive compounds, including peptides with significant therapeutic potential. Among the various bioactive peptides, inhibitory peptides have gained attention due to their ability to modulate essential biological processes [48]. Marine-derived peptides have unique structural characteristics due to the diverse environmental conditions of the marine ecosystem, contributing to their potent bioactivity [22,49]. Many of these peptides are derived from proteins found in various parts of marine organisms, including muscle, skin, bones, and even by-products that are often discarded as waste. The therapeutic potential of these peptides is being explored across various domains, with a particular focus on ACE inhibitors. ACE-inhibitory peptides are crucial in managing hypertension, a major risk factor for cardiovascular diseases [50]. These peptides exhibit strong ACE inhibition, which can help in reducing blood pressure by preventing the formation of angiotensin II, a potent vasoconstrictor (Figure 3) [51]. Table 1 provides a comprehensive summary of various marine peptides known to exhibit potential ACE-inhibitory properties derived from marine plants and animals.

The appeal of marine-derived ACE-inhibitory peptides lies in their bioactivity and relatively low risk of side effects compared to synthetic ACE inhibitors. Marine peptides are typically smaller and more easily absorbed by the body, and their natural origin reduces the likelihood of adverse reactions [105]. The extraction and isolation of ACE-inhibitory peptides from marine organisms require specific techniques to ensure the recovery of active compounds. The most common method involves enzymatic hydrolysis, where marine proteins are broken down by enzymes such as alcalase, pepsin, trypsin, and papain. A number of different approaches are being investigated for the production of bioactive peptides. These approaches include enzymatic hydrolysis, microbial fermentation, chemical hydrolysis, and genetic engineering strategies [106,107]. Although chemical hydrolysis is less selective, it is able to facilitate the quick breakdown of protein structures.

On the other hand, microbial fermentation may be very helpful for large-scale manufacturing. Because of its specificity and effectiveness in releasing target peptides with the necessary bioactivity, enzymatic hydrolysis continues to be the technique of choice for study, despite the fact that alternative approaches such as microbial fermentation, chemical hydrolysis, and genetic engineering have been investigated [108]. This process releases bioactive peptides, which are then isolated and purified through various chromatographic techniques [7,109]. The specificity of the protease and the circumstances under which the proteolysis takes place are two of the most important factors that determine the yield and functionality of the target peptides [110]. It is necessary to choose different enzymes in order to maximize the yield and activity of the peptides [111,112]. This is carried out in accordance with the source material. For example, the choice of protease may have an effect on the hydrolyzed peptide bonds, which can result in either the production of active ACE-inhibitory peptides or the synthesis of inactive fragments [113]. As a means of maximizing the generation of bioactive peptides while simultaneously reducing the breakdown of the active components, it is necessary to exercise precise control over conditions such as pH, temperature, and hydrolysis time. There is a possibility that the bioactive peptides that are needed cannot be produced if proper specificity and control are not present [107].

For example, fish proteins, such as those from skipjack tuna, *Alaska pollock*, and monkfish, are often hydrolyzed using a combination of alcalase, pepsin, and trypsin to yield potent ACE-inhibitory peptides [42,52,55]. Similarly, enzymatic hydrolysis is applied to mollusks, such as oysters and razor clams, and seaweeds like *Laminaria* and *Undaria* sp., to ACE-inhibitory peptides [91,92]. The resulting peptides are further purified using techniques such as ultrafiltration, gel permeation chromatography, and high-performance liquid chromatography (HPLC) to isolate the most active fractions. To create bioactive peptides with a high ACE-inhibitory activity, it is necessary to pay great attention to the enzymes used and optimize the circumstances under which the hydrolysis process takes place [114].

### 3.1. ACE-Inhibitory Peptides from Marine Animals

Fish are among the most extensively studied marine animals for the isolation of ACE-inhibitory peptides. Various parts of fish, including skin, bones, and muscles, have been shown to contain proteins that, when hydrolyzed, release peptides with potent ACE-inhibitory activity (Table 1) [115]. Peptides derived from the swim bladders of monkfish (*Lophius litulon*) and skin of shortfin scad (*Decapterus macrosoma*) have demonstrated significant inhibitory effects, with IC_50_ values of 0.63 mg/mL and 0.20 mg/mL, respectively [36,52]. Similarly, peptides from the dark muscle of skipjack tuna (*Katsuwonus pelamis*) and the bones of Atlantic salmon (*Salmo salar*) have been shown to exhibit strong ACE-inhibitory properties, with IC_50_ values as 87.11 µmol/L and 31.63 µmol/L [43,54]. These fish-derived peptides are typically obtained through enzymatic hydrolysis using enzymes such as alcalase, pepsin, and trypsin. The peptides produced are often small in size, making them easily absorbed in the human gastrointestinal tract. Studies have shown that these peptides not only inhibit ACE but also possess other health-promoting properties, such as antioxidant activity, further enhancing their therapeutic potential [70,73,85].

Mollusks, including species such as oysters (*Crassostrea gigas*), razor clams, and mussels, are also rich sources of ACE-inhibitory peptides. These peptides are often extracted from various parts of the mollusk, including the hepatopancreas, muscle, and shell, using enzymatic hydrolysis (Table 1) [78,79,80,112,116]. Peptides derived from the muscle of the deep-sea mussel (*Gigantidas vrijenhoeki*) and the hepatopancreas of *Apostichopus japonicus* have shown promising ACE-inhibitory activity, with IC_50_ values of 0.007 µmol/L and 333.5 µmol/L, respectively [78,86]. Furthermore, the extraction of peptides from mollusk byproducts, such as shells and waste material, offers a sustainable strategy to use marine resources that would otherwise go to waste [117]. Crustaceans, such as shrimp and crabs, are another important source of ACE-inhibitory peptides. For instance, peptides from the heads of kuruma shrimp (*Marsupenaeus japonicus*) have demonstrated strong ACE-inhibitory activity, with IC_50_ values of 125.58 μmol/L [87].

**Figure 4:**
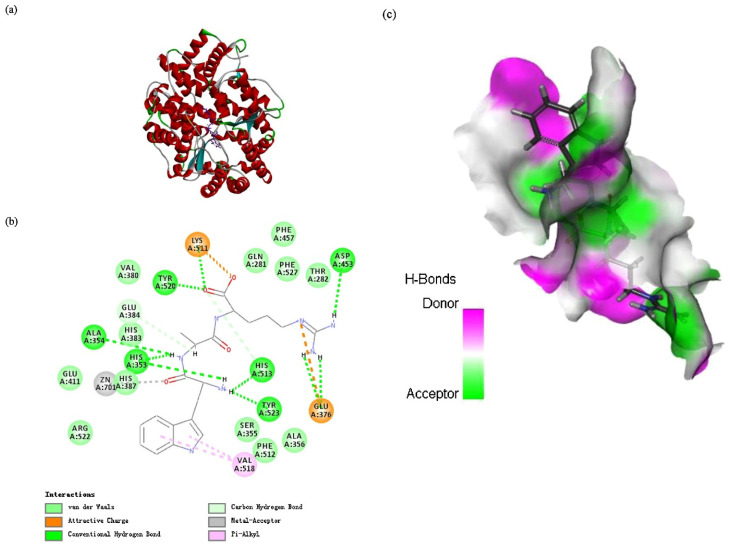
Docking analysis of WAR binding to ACE (PDB: 1O86), with interaction residues depicted in different colors. (**a**) 3D model of the ACE-WAR complex. (**b**) 2D interaction map between ACE and WAR. (**c**) 3D surface plot of hydrogen bonds formed by WAR at the binding site. Reprinted with permission from [63]. Copyright © 2020, Elsevier.

Molecular docking has become a powerful tool for understanding the interaction mechanisms between marine ACE-inhibitory peptides and their target enzyme, ACE. This computational technique allows researchers to predict how peptides bind to the active sites of ACE, providing insights into their inhibitory mechanisms and potential therapeutic efficacy [50]. The peptide WAR, derived from the swim bladder of monkfish (*Lophius litulon*), has been extensively studied using molecular docking techniques (Figure 4) [63]. The docking analysis revealed that WAR forms multiple hydrogen bonds with key amino acids in the ACE active site, including Ala354, Glu384, His353, and Lys511. These interactions suggest a stable binding conformation that likely contributes to WAR’s potent ACE-inhibitory activity. The peptide WAR also established interactions with ACE residues via both hydrogen bonds and hydrophobic interactions, particularly with Val518 and Thr701, indicating its strong binding affinity and non-competitive inhibitory mechanism. Furthermore, the study demonstrated that the ACE-peptide was stabilized using bonding energies, and ACE-WAR docking exhibited a strong binding affinity with ACE, with CDOCKER ENERGY values of −93.322 kcal/mol.

**Figure 5:**
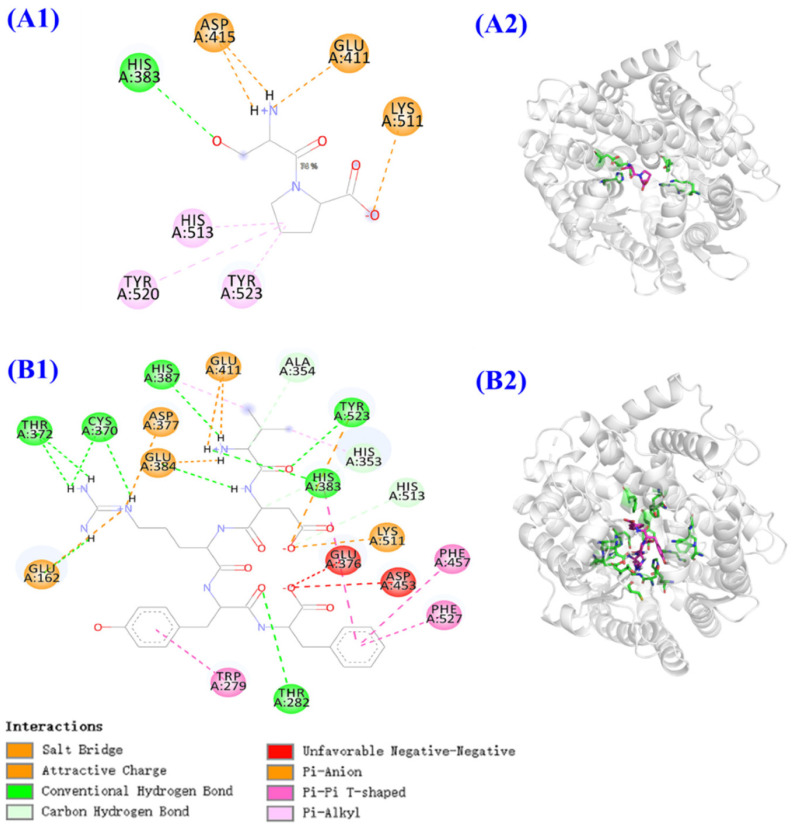
Molecular docking studies of TMAP1 and TMAP2 with ACE. (**A1**) Two-dimensional interaction map depicting TMAP1 binding to ACE. (**A2**) Three-dimensional representation of the interaction between TMAP1 and ACE. (**B1**) Two-dimensional interaction map showing ACE binding with TMAP2. (**B2**) Three-dimensional visualization of TMAP2 interacting with ACE. Reprinted from [69]. Copyright © 2022 by the authors. Licensee MDPI, Basel, Switzerland.

Similarly, peptides derived from the protein hydrolysate of skipjack tuna (*Katsuwonus pelamis*), such as TMAP1 and TMAP2, have been analyzed for their ACE-inhibitory potential (Figure 5) [69]. The docking studies on these two peptides (TMAP1 and TMAP2) have shown that these peptides interact effectively with ACE’s three major active site pockets (S1, S2, and S1’). The peptide forms hydrogen bonds with key residues like Ala354 and His353 and also engages in hydrophobic interactions with residues such as Tyr523 and Tyr520. These interactions not only stabilize the peptide within the ACE active site but also enhance its inhibitory potency. In addition, the molecular docking study showed that TMAP1 and TMAP2 showed an affinity for ACE that was −5.7 and −9.7 kcal/mol, respectively. These interactions stabilize the peptide within the ACE active site and enhance its inhibitory potency.

Moreover, the binding of LEPWR that is generated from Pacific saury to ACE results in structural rearrangements surrounding the zinc ion cofactor, which is essential for the catalytic activity of ACE itself [66]. The capacity of ACE to stabilize transition states during substrate processing is diminished due to LEPWR’s coordination with zinc, which disrupted the usual placement of zinc-dependent residues. Not only does this conformational change limit the enzymatic activity of ACE, but it also adds to the enzyme’s lower stability under physiological settings, which in turn allows it to catalyze processes with less efficiency [118].

Molecular dynamics simulations have further shown that the binding of peptides like TMAP and WAR to ACE reduces the flexibility of key loops near the active site, which limits substrate access [63,69]. This rigidity caused by peptide binding increases the overall structural stability of the peptide–ACE complex [119]. Thus, the conformational changes induced by marine-derived peptides result in a dual impact: they enhance the structural stability of the peptide–ACE complex, while simultaneously compromising the enzyme’s catalytic efficiency. This balance between stability and inhibition underscores the therapeutic potential of marine peptides, which offer a precise mechanism of action with a reduced risk of undesirable effects [120].

To provide a more comprehensive analysis, we now compare the binding of marine-derived ACE-inhibitory peptides to the well-characterized synthetic inhibitor, captopril. Captopril, a widely used ACE inhibitor, forms strong interactions with the zinc ion at the active site of ACE, coordinating with key residues such as Glu384 and His353 [121]. In contrast, many marine-derived peptides interact through hydrogen bonding and hydrophobic interactions with different residues in the active site. For instance, peptides like WAR and LEPWR from marine sources also bind to the zinc coordination site, mimicking the interactions of captopril but with slightly different sequence motifs that allow for competitive inhibition [63,66].

Further, the sequence motifs of marine peptides tend to feature hydrophobic residues, which promote stronger interactions with the S1 and S2 pockets of ACE [122]. This variation in binding modes between peptides and synthetic inhibitors like captopril offers an opportunity to develop novel inhibitors that may possess improved bioavailability or reduced side effects compared to existing pharmaceuticals.

### 3.2. ACE-Inhibitory Peptides from Marine Seaweeds

Algae-derived peptides have been extensively studied for their potential as natural ACE inhibitors (Table 1). For instance, Oarweed (*Laminaria digitata*), a brown algae species, has been found to contain ACE-inhibitory peptides with an IC_50_ value of 133.1 µg/mL, obtained through enzymatic hydrolysis using viscozyme and alcalase [92]. Another notable example is *Spirulina*, a microalga, which, when hydrolyzed using protease K, produces peptides with an IC_50_ value of 2.88 µmol/L [89]. Additionally, extracts from *Caulerpa lentillifera* exhibit an IC_50_ of 58.89 µg/mL when treated with trypsin, indicating the effectiveness of enzymatic hydrolysis in releasing bioactive peptides from algae [97]. These findings highlight the promise of algae as a source of potent ACE-inhibitory peptides, which could be utilized in functional foods aimed at managing hypertension.

**Figure 6:**
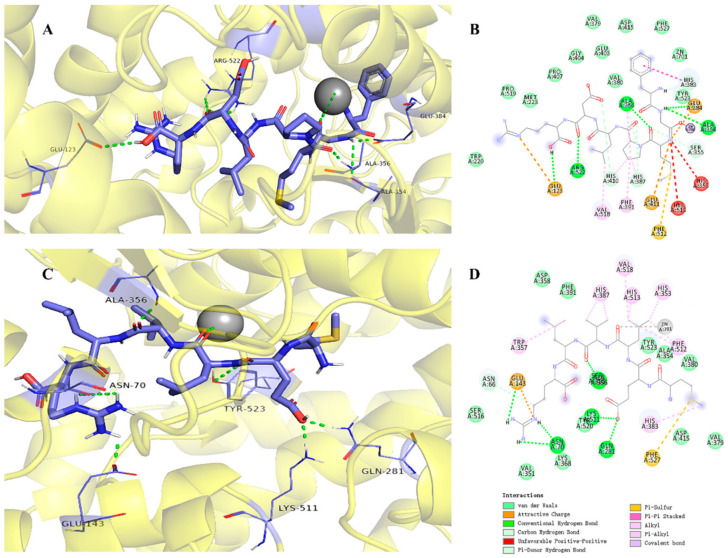
Molecular docking findings for FGMPLDR and MELVLR binding to ACE (PDB: 1O8A). (**A**) Three-dimensional view of the interaction between ACE and FGMPLDR. (**B**) Two-dimensional interaction map showing FGMPLDR with ACE. (**C**) Three-dimensional visualization of ACE interacting with MELVLR. (**D**) Two-dimensional interaction map illustrating the binding details of MELVLR with ACE. Reprinted from [95]. Copyright © 2019 by the authors. Licensee MDPI, Basel, Switzerland.

Marine algae have emerged as a promising source of ACE-inhibitory peptides, with molecular docking studies providing insights into the mechanisms by which these peptides interact with the ACE enzyme. These studies are crucial for understanding the structural basis of ACE inhibition and guiding the development of more effective inhibitors. For instance, the peptide FGMPFLDR, derived from the marine macroalga *Ulva intestinalis*, has been extensively analyzed using molecular docking techniques (Figure 6) [95]. The docking studies revealed that FGMPFLDR forms multiple hydrogen bonds with key residues in the ACE active site, including Ala354, Glu384, Ala356, and Asp522. Additionally, the peptide’s interaction with the ACE enzyme is stabilized by van der Waals forces, further enhancing its binding affinity. According to the molecular analysis results, the FGMPLDR interacts with the active site of the ACE in the presence of Zn(II), with binding energies of −2.78 kcal/mol. However, it was also observed that FGMPFLDR interacts with the S1’ pocket of ACE, specifically with Ala354 and Glu384, which might have contributed to a loss in ACE activity. The peptide’s interaction with Zn(II) ions, where Gly and Leu coordinate with the metal ion, may have influenced this reduction in activity, indicating a complex interaction that warrants further investigation. In addition to the previously mentioned peptides, the seaweed-derived peptide AKYSY, isolated from the algae *Laminaria japonica*, has been extensively studied for its potent ACE-inhibitory properties [103]. This peptide exhibits significant inhibition with an IC_50_ value of 2.42 μmol/L, making it one of the more effective peptides in seaweed research. Its effectiveness and wide recognition in ACE inhibition studies highlight its therapeutic potential in managing hypertension. In addition to the previously mentioned peptides, the seaweed-derived peptide AKYSY, isolated from the algae *Porphyra yezoensis*, has been extensively studied for its potent ACE inhibitory properties [103]. This peptide exhibits significant inhibition with an IC_50_ value of 1.52 μmol/L, making it one of the more effective peptides in seaweed research. Its effectiveness and wide recognition in ACE inhibition studies highlight its therapeutic potential in managing hypertension.

**Figure 7:**
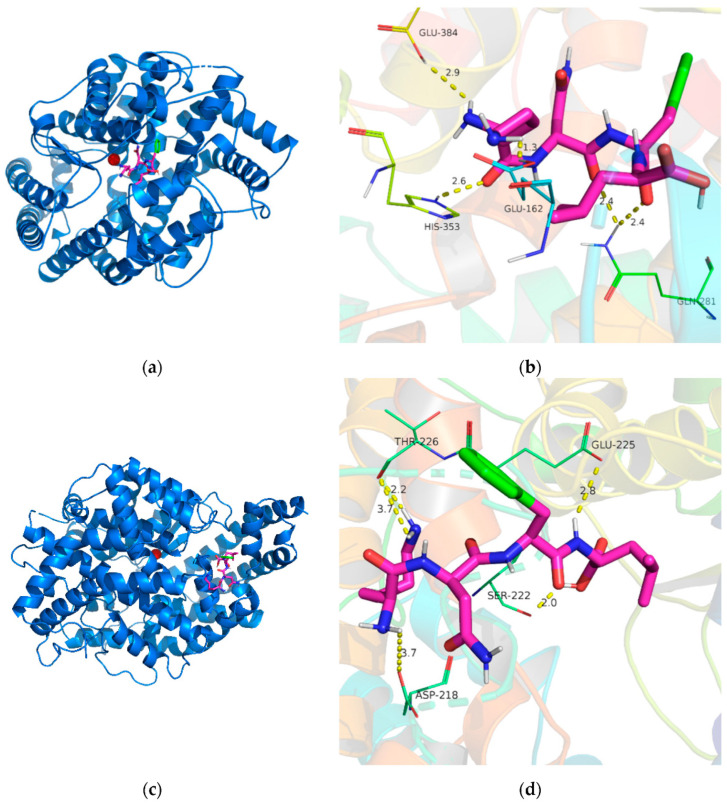
Molecular docking analysis of KNFL binding to ACE. (**a**) Visualization of KNFL (colored green) interacting with the active sites of ACE, depicted as a cartoon representation, with a zinc ion (colored red) highlighted at the active site. (**b**) Detailed interaction map showing KNFL (illustrated as sticks) and ACE residues (represented as lines), with hydrogen bonds indicated by yellow dashed lines. (**c**) Simulation of KNFL (green) interacting with non-active sites of ACE, also shown as a cartoon, with the zinc ion (red) at the active site. (**d**) Interaction details of KNFL (sticks) with ACE residues (lines), with hydrogen bonds marked by yellow dashed lines. Reprinted from [91]. Copyright © 2021 by the authors. Licensee MDPI, Basel, Switzerland.

Similarly, the peptide KNFL, isolated from *Undaria pinnatifida* (wakame), has been the subject of detailed molecular docking studies (Figure 7) [91]. These studies have shown that KNFL interacts with ACE’s active sites by forming hydrogen bonds with several critical residues, including Glu162, Gln281, His353, and Glu384. The peptide’s binding is also influenced by the shorter length of its hydrogen bonds, which increases the binding forces, particularly involving Glu and His residues. This kind of hydrogen interaction of KNFL peptide with the active and non-active sites of ACE exhibited binding affinity values of −6.9 kcal/mol and −5.6 kcal/mol, respectively. This interaction is crucial for the stabilization of the peptide–ACE complex, which inhibits the enzyme’s catalytic activity. KNFL’s binding to ACE is further strengthened by additional hydrogen bonds formed with residues such as Ser222, Thr226, Glu225, and Asp218, contributing to its effective inhibition of ACE. These molecular docking studies underscore the significance of hydrogen bonding, van der Waals interactions, and coordination with metal ions in the binding of algae-derived peptides to ACE [95]. By revealing the specific interactions at the molecular level, these studies provide valuable information for the design of new ACE-inhibitory peptides with enhanced potency.

## 4. Possible Extraction and Purification Approaches of ACE-Inhibitory Peptides

**Figure 8:**
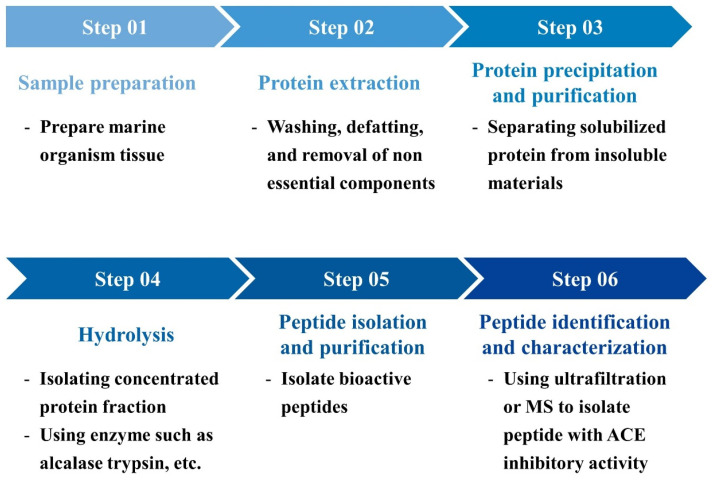
Flow diagram depicting the process of extracting and purifying ACE-inhibitory peptides from marine organisms. The diagram illustrates the key steps: sample preparation, initial extraction, precipitation, hydrolysis, chromatographic separation, purification, and final characterization. Each stage is designed to isolate peptides with potent ACE-inhibitory activity from marine sources, ensuring their potential for therapeutic applications.

The extraction and purification of ACE-inhibitory peptides from marine organisms is a complex process designed to maximize the yield and bioactivity of the peptides while preserving their structural integrity. This process typically begins with the careful preparation of marine tissue, followed by extraction, precipitation, purification, and, finally, the identification of the active peptides (Figure 8). This multi-step process requires the careful consideration of each phase to maximize the yield and bioactivity of the peptides [123].

This process typically starts with the preparation of the raw material. After preparing the raw material by cleaning and defatting, the proteins are extracted using solvents like acetic acid to ensure that they are fully dissolved while maintaining their integrity. The soluble proteins are then separated from insoluble material through filtration. Next, the proteins are concentrated by precipitation, typically using salts like NaCl, and then collected via centrifugation. These concentrated proteins undergo enzymatic hydrolysis, where enzymes such as alcalase break them down into smaller, bioactive peptides. The resulting mixture is purified using techniques like ultrafiltration and high-performance liquid chromatography (HPLC) to isolate the peptides with the highest ACE-inhibitory activity. Finally, the purified peptides are identified and characterized through mass spectrometry, revealing their molecular structure and confirming their potential as natural antihypertensive agents. This streamlined process is essential for obtaining potent ACE-inhibitory peptides that can be further developed into therapeutic applications [124,125,126,127].

In the study of peptides from *Lophius litulon* (monkfish) swim bladders, non-collagenous proteins were removed using NaOH, followed by defatting with butanol to eliminate lipids and thorough washing with cold distilled water to yield a clean, protein-rich material [52]. Similarly, in the extraction of peptides from *Oreochromis niloticus* (tilapia) skin, the tissue was cleaned and defatted to ensure high-quality protein extraction [65]. In both studies, the prepared tissue was treated with acetic acid to solubilize the proteins, which were then filtered to obtain a protein-rich solution ready for precipitation. Precipitation was achieved using NaCl for monkfish and ammonium sulfate for tilapia, followed by centrifugation to concentrate the proteins for further purification. The concentrated proteins were subjected to enzymatic hydrolysis using enzymes such as alcalase, which resulted in the formation of smaller peptides with ACE-inhibitory properties. These peptides were then purified through ultrafiltration and HPLC to isolate those with the strongest ACE inhibition potential. Subsequently, mass spectrometry and LC–MS/MS were employed to identify and characterize the peptides, verifying their sequences and biological activity. This methodical process underscores the effective extraction and purification of ACE-inhibitory peptides from marine sources, showcasing their potential as natural antihypertensive agents for incorporation into functional foods and nutraceuticals.

## 5. In Vivo and Clinical Studies

The investigation of ACE-inhibitory peptides derived from marine organisms has extended into in vivo studies, where their potential antihypertensive effects have been evaluated using animal models. These studies offer valuable insights into the practical application of these peptides in lowering blood pressure and addressing hypertension-related health issues (Table 2). In the evaluation of ACE-inhibitory peptides derived from marine organisms, the spontaneously hypertensive rat (SHR) model is the most commonly used as an in vivo model [128]. This model is particularly valuable due to its genetic predisposition to develop hypertension, closely mimicking the essential hypertension observed in humans. In SHR models, ACE-inhibitory peptides are administered to assess their potential to lower blood pressure over time [129,130].

One of the significant findings comes from the study of a peptide named LEPWR, derived from the muscle tissue of Pacific saury [66]. In rat models, this peptide demonstrated noteworthy antihypertensive activity. When administered at a dosage of 2000 mg/kg, LEPVR significantly reduced systolic blood pressure (SBP), suggesting its effectiveness as a natural antihypertensive agent. Similarly, a peptide derived from tilapia, QAGLSPVR, has shown promising results [131]. The QAGLSPVR peptide, obtained from the skin of *Oreocharis niloticus*, also showed significant reductions in SBP and diastolic blood pressure (DBP) in rat models. Moreover, this peptide led to a decrease in serum ACE activity, further supporting its potential in managing hypertension. Research into a peptide from *Takifugu flavidus*, PPLLFAAL, also highlights its antihypertensive efficacy [53]. These peptides, when administered at doses of 5 mg/kg, resulted in significant reductions in SBP. Notably, PPLLFAAL was particularly effective in reducing the maximum SBP observed in these models.

Marine invertebrates, such as *Apostichopus japonicus* and scallop *Chlamys farreri*, have also been explored as sources of antihypertensive peptides [18,86]. The peptide HDWWKER from *A. japonicus* and GAWA from *C. farreri* were tested at 12 and 50 mg/kg doses in rat models, respectively. These peptides reduced SBP and influenced the expression of genes related to the RAS in the kidneys, demonstrating a comprehensive approach to blood pressure regulation. Several clinical investigations are underway to translate these results from animal models to human applications. A peptide produced from *Styela clava* was provided to human participants in capsule form at a dose of 500 mg/day, resulting in a substantial drop in both SBP and DBP [132]. Another clinical trial using a peptide from *Katsuwonus pelamis* (LKP) at 5 mg/day found a substantial decrease in blood pressure and angiotensin II levels [133]. Additionally, a peptide isolated from shrimp (*Pandalus borealis*) injected at 1200 mg resulted in significant blood pressure decreases [134]. These findings show that marine peptides may have significant promise for reducing hypertension in people, but further large-scale clinical studies are required to prove this.

While the in vivo findings are promising, there are important limitations to consider before translating these results into human clinical applications. A major challenge lies in the bioavailability of these peptides when administered orally or through other therapeutic routes. Peptides are often susceptible to degradation by digestive enzymes, reducing their effectiveness in human systems [135]. Furthermore, the stability and specificity of these peptides in targeting ACE activity within complex human physiology require thorough evaluation [136]. Clinical studies investigating the efficacy, safety, and long-term effects of these peptides in humans are still lacking. This gap needs to be addressed, with a particular focus on ensuring that these peptides maintain their antihypertensive properties without adverse side effects in humans. Addressing these limitations is crucial for advancing these findings from preclinical studies to therapeutic applications [137,138].

For scientific research, isolating single peptides is often necessary to understand their specific bioactivity and their mechanisms of action [139]. However, when it comes to applied uses such as therapeutic or nutraceutical formulations, there is an ongoing debate about whether preparations containing mixtures of peptides or a single peptide with maximum activity are more effective [140]. From a practical standpoint, mixtures of peptides may provide synergistic effects, where different peptides could target multiple pathways, potentially enhancing overall efficacy. These mixtures might also have improved bioavailability or broader therapeutic applications compared to single peptides [141]. Moreover, producing peptide mixtures can be more cost-effective, as expensive isolation and purification methods may not be required [107]. This is particularly beneficial for industrial-scale production, where minimizing costs while maintaining efficacy is a key concern. Purification methods like chromatography, though effective for isolating single peptides, can significantly increase production costs [142]. Thus, using crude or semi-purified peptide mixtures may provide a more viable approach for large-scale applications, especially when the mixture still exhibits potent bioactivity. Further studies are needed to compare the long-term efficacy, safety, and economic benefits of single peptides versus peptide mixtures in real-world applications. Balancing these factors will be essential for optimizing the development of peptide-based products for therapeutic and nutraceutical markets [143].

**Table 2:** In vivo and clinical studies of marine-sourced ACE-inhibitory peptides.

Name	Tissue	IC_50_	Peptide Sequence	Animal Model	Concentration	Action and Role	Reference
Mackerel pike (*Pacific saury*)	muscle	99.5 μmol/L	LEPWR	Spontaneously hypertensive rat	2000 mg/kg BW	- Reduced SBP from 181 mmHg to 168.5 mmHg, with maximum effect observed at 8 h	[66]
Nile tilapia (*Oreochromis niloticus*)	muscle	2.577 μmol/L	LSGYGP	SHR	10 mg/kg BW	- Significantly reduced SBP in SHRs by 20 mmHg, with the effect noticeable 2 h post-administration and maintained for 9 h	[144]
Yellowbelly pufferfish (*Takifugu flavidus*)	muscle	0.58 mg/mL	PPLLFAAL	SHR	5 mg/kg BW	- SBP reduction from 193 to 145 mmHg at 4 h which then gradually recovered to 154 mmHg at 24 h - DBP decreased significantly from 135 to 107 mmHg at 4 h and then recovered to 113 mmHg at 24 h	[53]
*Sargassum maclurei*	-	72.24 μmol/L	RWDISQPY	SHR	150 mg/kg BW	- Significantly reduced both DBP and SBP in SHRs from the second week	[145]
Manila clam (*Ruditapes philippinarum*)	muscle	1.37 μmol/L	TYLPVH	SHR	10.0 mg/kg BW	- A maximum SBP reduction of 26.47 mmHg at 6 h	[77]
Flounder	muscle		MEVFVP	SHR	40 mg/kg BW	- Decrease in SBP between 3–6 h and reduced ET-1 mRNA expression, plasma levels of ET-1, angiotensin II, and aldosterone	[146]
Yellowbelly pufferfish (*Takifugu flavidus*)	muscle	93.5 μmol/L	TLRFALHGME	SHR	4 mg/kg BW	- Notable reduction in SBP was observed between 2 and 8 h, with the lowest recorded SBP of 171 mmHg at 4 h - By 8 h, the SBP gradually increased, returning to 190 mmHg	[60]
Manila clam (*Ruditapes philippinarum*)	-		-	DOCA–salt hypertensive rats	400 mg/kg BW	- Both systolic and diastolic blood pressure significantly decreased within one week - Systolic blood pressure normalized after five weeks	[147]
Leathery sea squirt (*Styela clava*)	-	16.48 μmol/L	LWHTH	Spontaneously hypertensive rat (SHR)	40 mg/kg BW	- Significantly reduced both SBP and DBP with maximum reductions observed at 3 h The SBP decreased by 89.4% from 207.6 mmHg to 185.4 mmHg, and DBP showed a similar reduction trend	[85]
Sea cucumber (*Apostichopus japonicus*)	gonad	583.6 μmol/L	HDWWKER	SHR	12 mg/kg	- Significantly reduced SBP between 2 and 8 h, with the lowest SBP of 176 mmHg occurring at 4 h	[86]
Seahorse (*Hippocampus abdominali*s)	-	0.088 mg/mL	CNVPLSP	SHR	100 mg/kg BW	- Significantly decreased the SBP by the action of ACE inhibition	[71]
Oyster (*Crassostrea gigas*)	-	4.287 mmol/L	AEYLCEAC	SHR	15 mg/kg BW	- In a 12 h trial, significantly lower SBP and DBP, with the lowest values at 3 h - The 4-week trial also showed downregulated Ren1 and Agtr1 gene expression and upregulated Adrb3 in rats’ kidneys	[79]
Marine fish Cobia (*Rachycentron canadum*)	skin	0.51 μmol/L	IWW	SHR	56.7 mg of peptide/BW	- Significant decrease in SBP by 21.9 mmHg and DBP by 15.5 mmHg within 2 to 8 h, with levels returning to baseline after 24 h	[148]
Nile tilapia (*Oreochromis niloticus*)	skin	68.35 μmol/L	QAGLSPVR	SHR	20 mg/kg BW	- Significant decrease in both SBP and DBP, with reductions of 41.86 mmHg and 40.40 mmHg, respectively, observed 3 h post-administration	[131]
Cuttlefish (*Sepia officinalis*)	muscle	5.22 μmol/L	VELYP	SHR	10 mg/kg BW	- Significantly reduced SBP and DBP, with the most potent SBP reduction (20 mmHg) occurring at 6 h and the maximal DBP reduction (14.7 mmHg) at 2 h.	[149]
Scallop (*Chlamys farreri*)	skirt	0.74 mg/mL	AGFAGDDAPR	SHR	50 mg/kg	- A maximum SBP reduction of 56.19 mmHg and DBP reduction of 15.43 mmHg at 6 h after a single administration, and even more pronounced effects after 8 weeks of continuous administration	[18]
Pearl oyster (*Pinctada fucata*)	muscle	109.25 μmol/L	GWA	SD rat	10 mg/kg BW	- Significant reductions in SBP and DBP, with a maximum SBP decrease of 16.7 mmHg and DBP decrease of 20.7 mmHg after 20 min	[116]
Manila clam (*Ruditapes philippinarum*)	meat	8.16 μmol/L	VISDEDGVTH	SHR	8 mg/kg	- Reduced SBP by 22.1% and DBP by 18.6% at 8 h post-gavage, with blood pressure returning to baseline after 24 h - Over an 8-week trial, RBPs led to a 30.0% decrease in SBP and a 29.4% decrease in DBP	[150]
*Gracilariopsis lemaneiformis*	-	9.64 μmol/L	CILR	SHR	10 mg/kg BW	- Reduced SBP showing a greater reduction (34 mmHg) from 204 to 170 mmHg at 2 h significantly reduced DBP from 145 to 118 mmHg at 2 h	[151]
*Styela clava*	flesh	-	-	Human	500 mg/day, capsule	- Significant decrease in both SBP and DBP	[132]
*Katsuwonus pelamis*	-	2.4 μg/mL	LKPNM	Human	5 mg/day	- Significant decrease in blood pressure	[133]
Shrimp (*Pandalus borealis*)	-	-	-	Human	1200 mg	- Angiotensin II levels were significantly reduced relative to baseline	[134]

## 6. Conclusions and Future Perspectives

In conclusion, marine ACE-inhibitory peptides provide promise for antihypertensive drug development. Through its RAS and KKS activities, ACE regulates blood pressure and fluid balance, making it a key hypertension target. According to this review, bioactive peptides from fish, mollusks, and algae suppress ACE. Marine-derived peptides have fewer side effects than synthesized ACE drugs. We can now identify bioactive chemicals from marine resources using enhanced peptide-extraction and -purification methods. Enzymatic hydrolysis and chromatographic purification work well to develop strong ACE inhibitors. Additionally, molecular docking studies have illuminated the interaction processes between these peptides and ACE, helping to build more potent inhibitors. Chemical synthesis can effectively synthesize short ACE-active peptides; thus, other methods should be considered. Chemical synthesis allows for peptide purity, sequence control, and scalability, although it may cost more than seawater extraction. Marine-derived peptides typically have effects beyond ACE inhibition, which chemically generated peptides may lack. Thus, future studies should assess both techniques’ long-term cost-efficiency, effectiveness, and therapeutic advantages.

Marine-derived peptides reduce blood pressure and modulate RAS components in animal models, proving their antihypertensive potential. These data suggest that these peptides may treat hypertension and associated cardiovascular problems. Before these peptides may be used clinically, safety, bioavailability, and effectiveness must be studied in humans. The search for new ACE inhibitors in marine species helps produce safer and more effective hypertension medications and meets the rising desire for natural and sustainable therapies. As the study continues, these marine-derived peptides may improve cardiovascular health and provide alternative hypertension treatments. In the context of hypertension treatment and therapeutic development, various interesting future perspectives may be envisioned based on the present breakthroughs in ACE-inhibitory peptides obtained from marine organisms. These include the following:

1. Research on marine species as a source of bioactive peptides is still in its infancy. The vast and unknown marine life holds enormous potential for discovering novel ACE-inhibitory peptides. Underused marine species should be studied to uncover new peptide candidates with greater medicinal value;
2. To increase the synthesis and bioactivity of ACE-inhibitory peptides, novel extraction and purification procedures are required. Future research might include high-throughput screening, novel hydrolysis enzyme systems, and improved chromatographic methods. These advancements may enhance industrial scalability, separation efficiency, and peptide purity;
3. Detailed structural investigations, such as X-ray crystallography and NMR spectroscopy, are necessary to comprehend the interaction processes between ACE and marine-derived peptides. Future research should clarify these peptides’ binding interactions, conformational changes, and dynamics. This understanding will help create peptides with better binding affinity and specificity, possibly improving antihypertensive drugs;
4. In vivo animal studies have shown encouraging results, but human clinical trials are necessary to validate marine-derived peptides’ safety, effectiveness, and bioavailability. Future research should emphasize clinical studies to investigate these peptides’ long-term effects, appropriate dose regimes, and drug interactions. Successful clinical validation might allow these peptides to be used in mainstream therapy;
5. Marine-derived peptides may improve customized hypertension treatment. In future studies, peptide-based drugs might be tailored to genetic profiles, clinical issues, and lifestyle variables. Selecting peptides that fit an individual’s physiological demands may enhance treatment success and reduce negative effects;
6. Marine-derived ACE-inhibitory peptides for functional foods and nutraceuticals are becoming popular. Future supplements, fortified meals, and beverages should include peptides. Research should enhance these peptides’ stability and bioavailability in food to ensure efficacy and consumer appeal;
7. Addressing sustainability and environmental impacts is critical as marine-derived peptide demand increases. Sustainable harvesting, aquaculture for peptide manufacture, and marine by-product usage are needed to decrease waste. Making peptide extraction and manufacturing eco-friendly will help this method to persist;
8. Regulatory and legislative frameworks are essential for marine-derived peptide clinical and industrial uses. Future regulatory studies should advocate peptide-based product safety, efficacy, and quality control. Clear regulatory pathways will help innovative therapies become approved.

## Figures and Tables

**Table 1:** Sources of ACE-inhibitory peptides from diverse marine organisms.

Organism Type	Specific Species	Part Used	Extraction Method	Peptide Sequence	IC_5_0	Reference
Fish	Monkfish (*Lophius litulon*)	Swim bladders	Double-enzyme system (Alcalase + Neutrase)	SEGPK	0.63 mg/mL	[52]
Fish	Yellowbelly pufferfish (*Takifugu flavidus*)	Skin, meat	Enzymatic hydrolysis (Alcalase, pepsin, and trypsin)	PPLLFAAL	28 μmol/L	[53]
Fish	Shortfin scad (*Decapterus macrosoma*)	Bones, skins, and tails	Enzymatic hydrolysis (Alcalase)	RGVGPVPAA	0.20 mg/mL	[36]
Fish	Skipjack tuna (*Katsuwonus pelamis*)	Dark muscle	Enzymatic hydrolysis (Protease)	FPPDVA	87.11 μmol/L	[54]
Fish	Alaska pollock (*Gadus chalcogrammus*)	Skins	Enzymatic hydrolysis (Alcalase, trypsin)	GPLGVP	105.8 μmol/L	[55]
Fish	Skipjack tuna (*Katsuwonus pelamis*)	Blood	Enzymatic hydrolysates (Neutrase)	-	0.19 mg/mL	[56]
Fish	Atlantic salmon (*Salmo salar* L.)	Skin	Enzymatic hydrolysis (Alcalase)	GR	0.73 mg/mL	[57]
Fish	Mackerel (*Scomber japonicus*)	Muscle	Enzymatic hydrolysis (Papain)	PLITT	48.78 μmol/L	[58]
Fish	Large yellow croaker (*Larimichthys crocea*)	Muscles of the back	Enzymatic hydrolysis (Papain, trypsin)	IPYADFK	0.64 μmol/L	[59]
Fish	Yellowbelly pufferfish (*Takifugu flavidus*)	Skin	Enzymatic hydrolysis (Alcalase)	TLRFALHGME	93.5 μmol/L	[60]
Fish	Redwing sea robin (*Lepidotrigla microptera*)	Muscle	Enzymatic hydrolysis (Alcalase)	DLTAGLLE	0.13 mg/mL	[61]
Fish	Skipjack tuna (*Katsuwonus pelamis*)	-	Enzymatic hydrolysis (Alcalase)	ICY	0.48 mg/mL	[62]
Fish	Large yellow croaker (*Larimichthys crocea*)	-	-	WAR	31.2 μmol/L	[63]
Fish	Atlantic salmon (*Salmo salar*)	Bones	Enzymatic hydrolysis (Trypsin)	FCLYELAR	31.63 μmol/L	[43]
Fish	Cutlassfish (*Trichiurus lepturus*)	Muscle	Enzymatic hydrolysis (Pepsin)	FSGGE	0.033 mg/mL	[64]
Fish	Skipjack tuna (*Katsuwonus pelamis*)	Roe	Enzymatic hydrolysis (Flavorzyme)	YSHM	0.49 mg/mL	[42]
Fish	Nile tilapia (*Oreochroma niloticus*)	Skin	Enzymatic hydrolysis (Alcalase)	VGLFPSRSF	61.43 μmol/L	[65]
Fish	Pacific saury (*Cololabis saira*)	Muscle	Enzymatic hydrolysis (Neutrase)	LEPWR	99.5 μmol/L	[66]
Fish	Large head hairtail (*Trichiurus lepturus*)	-	Enzymatic hydrolysis (Alcalase)	QGPIGPR	81 μmol/L	[67]
Fish	Nile tilapia (*Oreochroma niloticus*)	Skin	Enzymatic hydrolysis (Purified from the hepatopancreas of Pacific white shrimp (*Litopenaeus vannamei*))	ARTCR	77.0 μmol/L	[68]
Fish	Skipjack tuna (*Katsuwonus pelamis*)	Muscle	Enzymatic hydrolysis (Alcalase)	SP	0.06 mg/mL	[69]
Fish	Mozambique tilapia (*Oreochromis mossambicus*)	Skin	Enzymatic hydrolysis (Papain)	GPLGAL	117.20 μmol/L	[70]
Fish	Big-belly seahorse (*Hippocampus abdominalis*)	-	Enzymatic hydrolysis (Protease)	CNVPLSP	0.088 mg/mL	[71]
Fish	Skipjack tuna (*Katsuwonus pelamis*)	Dark muscles	Enzymatic hydrolysis (Neutrase)	MKKS	0.269 mg/mL	[72]
Fish	European pilchard (*Sardina pilchardus*)	-	Enzymatic hydrolysis (Alcalase)	KFL	0.66 mg/mL	[73]
Fish	Stone fish (*Actinopyga lecanora*)	Muscle	Enzymatic hydrolysis (Bromelain)	ALGPQFY	0.012 mmol/L	[74]
Fish	Tuna (*Thunnus thynnus*)	Muscle	Enzymatic hydrolysis (Neutrase, alkaline)	LTGCP	64.3 μmol/L	[75]
Fish	Flounder (*Paralichthys olivaceus*)	Muscle	Enzymatic hydrolysis (Flavourzyme, kojienzyme, and protamex)	VFSGWAA	27.50 μg/mL	[76]
Mollusk	Manila clam (*Ruditapes philippinarum*)	Muscle	-	TYLPVH	1.37 μmol/L	[77]
Mollusk	Deep-sea mussel (*Gigantidas vrijenhoeki*)	Muscle	Enzymatic hydrolysis (Pepsin)	KLLWNGKM	0.007 μmol/L	[78]
Mollusk	Oyster (*Crassostrea gigas*)	Muscle	Enzymatic hydrolysates (In vitro gastrointestinal digestion)	AEYLCEAC	4.287 mmol/L	[79]
Mollusk	Razor clam (*Siliqua patula*)	-	Enzymatic hydrolysis	SCCGY	0.009 mmol/L	[80]
Mollusk	Akoya pearl oyster (*Pinctada fucata*)	Pearl	Enzymatic hydrolysis (Trypsin)	KKCHFWPFPW	4.17 μmol/L	[81]
Mollusk	Akoya pearl oyster (*Pinctada martensii*)	Pearl	Enzymatic hydrolysis (Pineapple protease, neutral protease)	AHYYD	2.102 mmol/L	[82]
Mollusk	Akoya pearl oyster (*Pinctada fucata*)	Pearl	-	KKCH	413.2 μmol/L	[83]
Sea horse	Flat-faced seahorse (*Hippocampus trimaculatus*)	-	Enzymatic hydrolysis (Alcalase)	PAGPRGPA	7.90 μmol/L	[84]
Tunicate	Leathery sea squirt (*Styela clava*)	-	Enzymatic hydrolysis (Pepsin)	LWHTH	16.42 μmol/L	[85]
Sea cucumber	Japanese spiky sea cucumber (*Apostichopus japonicus*)	Gonads	Enzymatic hydrolysis (Alcalase)	DDQIHIF	333.5 μmol/L	[86]
Crustacean	Kuruma Shrimp (*Marsupenaeus japonicus*)	Heads	Enzymatic hydrolysis (Papain)	ARL/I	125.58 μmol/L	[87]
Algae	Hudson (*Laminaria digitata*)	-	Enzymatic hydrolysis (Viscozyme)	-	590 μg/mL	[88]
Algae	*Spirulina* sp.	-	Enzymatic hydrolysis (Protease k)	TVLYEH	2.88 μmol/L	[89]
Algae	*Acrochaetium* sp.	-	Enzymatic hydrolysis	VGGSDLQAL	433.1 μmol/L	[90]
Algae	Wakame (*Undaria pinnatifida*)	-	Enzymatic hydrolysis (Bromelain)	KNFL	0.12 mg/mL	[91]
Algae	Oarweed (*Laminaria digitata*)	-	Enzymatic hydrolysis (Viscozyme, alcalase)	YIGNNPAKGGLF	133.1 µg/mL	[92]
Algae	Branched string lettuce (*Ulva prolifera*)	-	Enzymatic hydrolysis (Alcalase, papain)	DIGGL	10.32 μmol/L	[93]
Algae	*Pyropia pseudolinearis*	Leaves	Enzymatic hydrolysis (Thermolysin)	LRM	0.15 μmol/L	[94]
Algae	Sea lettuce (*Ulva intestinalis*)	-	Enzymatic hydrolysis (Trypsin)	FGMPLDR	219.35 μmol/L	[95]
Algae	Purple laver (*Porphyra dioica*)	-	Enzymatic hydrolysis (Alcalase flavourzyme)	TYIA	89.7 μmol/L	[96]
Algae	Sea grapes (*Caulerpa lentillifera*)	-	Enzymatic hydrolysis (Thermolysin)	FDGIP	58.89 μmo/L	[97]
Algae	*Bangia fuscopurpurea*	-	Enzymatic hydrolysis (Trypsin, pepsin)	ALLAGDPSVLEDR	57.2 μg/mL	[98]
Algae	*Gracilariopsis lemaneiformis*	-	Enzymatic hydrolysis (Trypsin)	QVEY	474.36 μmol/L	[99]
Algae	*Enteromorpha clathrata*	-	Enzymatic hydrolysis (Alcalase)	PAFG	0.014 mg/mL	[100]
Algae	*Palmaria palmata*	-	Enzymatic hydrolysis (Thermolysin)	LRY	0.01 mg/mL	[101]
Algae	*Ulva prolifera*	-	Enzymatic hydrolysis (Protease)	KAF	0.63 μmol/L	[102]
Algae	*Pyropia yezoensis*	-	Enzymatic hydrolysis (Pepsin)	AKYSY	1.52 μmol/L	[103]
Microalgae	*Isochrysis zhanjiangensis*	-	Enzymatic hydrolysates (In vitro gastrointestinal digestion)	FEIHCC	61.38 μmol/L	[104]
